# Better together? Effects of task complexity and social setting on performance and cognitive load

**DOI:** 10.3389/fpsyg.2026.1766737

**Published:** 2026-03-27

**Authors:** Marloes L. Nederhand, Floris Kervers, Leandra Fitriyadi, Isa Noordover, Bjorn B. De Koning

**Affiliations:** 1 Department of Psychology, Education and Child Studies, Erasmus School of Social and Behavioural Sciences, Erasmus University Rotterdam, Rotterdam, Netherlands; 2 Southampton Education School, University of Southampton, Southampton, United Kingdom

**Keywords:** cognitive load, efficiency, online collaboration, performance, task complexity

## Abstract

Framed within Cognitive Load Theory, this study investigated the effects of task complexity on performance, cognitive load, and efficiency on performance and cognitive load in an online learning environment. Using a between-subjects design, university students (*n* = 85) completed a set of tasks varying in complexity (low vs. high) either individually or in a group. It built on previous studies showing that task complexity and cognitive load significantly influence the efficiency of individual and group learning in an offline setting—groups perform better than individuals on high-complexity tasks, while individuals perform better than groups on low-complexity tasks. Results show higher performance, lower cognitive load, and higher efficiency on low-complexity than on high-complexity tasks. Surprisingly, collaboration improved performance on low-complexity tasks, whereas it reduced performance on high-complexity tasks. These results emphasize the importance of validating educational principles in online educational settings that are originally tested in offline settings. Ultimately, these findings can contribute to the optimization of online collaborative learning and the development of efficient instructions that balance both performance and cognitive load.

## Introduction

Online education has become an indispensable part of today’s higher education environment. Many courses contain elements of online education, ranging from instructional materials (e.g., videos) students can access online to interactive platforms they can use to discuss the course content. The vast majority of studies on online education focus on asynchronous learning, in which instructional materials are studied individually (Paudel, 2021). However, an essential goal of higher education is to effectively prepare students for collaborating on ultra-complex societal problems (i.e., wicked problems). These problems are naturally resistant to a clear definition and an agreed solution, which makes them difficult to solve alone (Head and Alford, 2013) and thus require collaborative effort. There exists a large research base indicating that collaboration impacts performance on complex tasks (e.g., Kirschner et al., 2011). For example, collaboration enables processing more information, enhances problem-solving, increases students’ motivation to achieve a goal, and helps to construct meaning from instructional materials (Hammar Chiriac, 2014; Kirschner et al., 2011). A key finding regarding collaborative learning in offline learning contexts is that collaboration is more beneficial than individual work on complex tasks, whereas for low complexity tasks individual students outperform collaborative groups, which is referred to as the collective working memory effect (Kirschner et al., 2011).

However, results obtained in an offline context do not necessarily translate to an online educational setting. When comparing online and offline educational settings, teachers indicate that they experience more constraints when discussing course content online with a group of students and see less social engagement amongst students when they need to work together (Verstraeten et al., 2025). It appears that the transaction costs associated with solving problems in an online environment are considerably higher than in an offline environment. This may be particularly relevant when collaborating on complex tasks because in this situation it is unclear whether the benefits of collaboration still outweigh transaction costs in an online environment. As online learning becomes increasingly mainstream, it is crucial to understand whether the same principles apply in online settings in order to determine if—and for which tasks—it is best used in higher education. The current study therefore experimentally examines whether social setting (i.e., working individually or together) in an online learning environment significantly impacts task performance on tasks that differ in complexity.

### The collective working memory effect

The collective working memory effect is typically explained from a Cognitive Load Theory (CLT) (Sweller et al., 2019) perspective. CLT is an instructional design theory built on the premises of human cognitive information-processing capabilities. At the core, it distinguishes between a long-term memory system that has unlimited capacity for storing information and a working memory system that is limited in both capacity and duration when processing information (Cowan, 2020). A consequence of working memory only being able to process a limited number of information elements simultaneously, is that when cognitive demands become too high, learning processes and performance can be compromised (Sweller et al., 2019). In that regard, the three types of cognitive load that CLT distinguishes come into play. Intrinsic cognitive load refers to the complexity of the information that is processed and is determined by the number of interacting elements in a task and/or the prior knowledge of the learner. Extraneous cognitive load relates to processing that does not contribute to learning and is caused by external factors, such as inadequate instruction methods or distractions in the environment. Germane cognitive load is considered a useful form of cognitive load as it contributes to schema construction. In the most recent conceptualization of CLT, germane cognitive load is assumed to not impose a load on its own but rather makes sure that working memory resources are spent on intrinsic aspects of the learning task so that cognitive activities are employed that are directly relevant to learning (Sweller et al., 2019). Together, CLT argues that to promote learning, it is important to optimize cognitive load in such a way that non-relevant cognitive processing (extraneous cognitive load) is minimized, relevant cognitive processing (germane cognitive load) is optimized, and the complexity of the task (intrinsic cognitive load) is kept within manageable limits (van Merriënboer et al., 2006).

According to CLT, the collective working memory effect is an example of how individual working memory capacity and duration limitations can be overcome by group or collaborative learning (Kirschner et al., 2011). As argued by Kirschner et al. (2009); also see (Janssen et al., 2010), a group of students can be seen as an information processing system that consists of several different working memories, and when working together, they create a collective working memory. In this way, the cognitive effort required to solve a learning task, can be divided among the group members who collectively have a larger cognitive capacity, reducing the individual intrinsic cognitive load (Kirschner et al., 2011, 2018). As a result, the available collective working memory can lead to a more efficient learning process for students who work together, yielding higher-quality schemas in their long-term memory, compared to students who work individually.

However, collaboration is not free of costs. Collaboration induces specific transaction costs that are not present when working individually. Specifically, collaboration requires coordination and attunement with each other about the (steps in a) task or about the way of working, all of which likely introduces extraneous cognitive load as these transaction costs are needed but do not directly contribute to learning or problem solving (Janssen and Kirschner, 2020). This might not be problematic if intrinsic cognitive load is low (e.g., when collaborating on a low-complexity task) but can hinder learning if intrinsic cognitive load is high (e.g., when collaborating on a complex task). Also, if transaction costs are higher than the intrinsic cognitive load generated by the task, the learning process can become inefficient and potentially hinder the learning process and performance (Retnowati et al., 2018).

### Empirical research on the collective working memory effect

Empirical research on the collective working memory effect is relatively limited and has yielded mixed results. The effect was established in a study by Kirschner et al. (2011), where participants were required to solve low-complexity and high-complexity problem-solving tasks either individually or collaboratively in a group. Results showed that groups performed more efficiently on high-complexity tasks than on low-complexity tasks, whereas the opposite was found when solving the tasks individually. Partially supporting findings were reported by Sankaranarayanan et al. (2021) who demonstrated that learners working collaboratively on high-complexity tasks performed better than students working individually. Similarly, work by Zambrano et al. (2019) indicates that on high-complexity tasks, learners with limited prior knowledge obtain higher retention performance, also after a delay, when collaborating than when working individually, although this occurred with higher reported cognitive load.

However, other research showed that the collective working memory effect does not occur under certain circumstances. According to studies by Retnowati et al. (2010, 2016) the type of task impacts whether the collective working memory effect is found. In their studies, participants had to solve low-complexity and high-complexity worked examples either individually or collaboratively. They found that, unlike the Kirschner et al. (2011) study, individuals outperformed groups on high-complexity tasks and that collaboration in groups proved more advantageous than individual work overall. This led the authors to conclude that when solving worked examples, rather than traditional problem-solving tasks, collaboration does not offer a benefit over individual learning on low- or high-complexity tasks. Another factor influencing the likelihood of finding the collaborative learning effect regards learners’ prior knowledge as evidenced by lower task performance and higher cognitive load when learners with high prior knowledge collaborated on complex tasks (Zambrano et al., 2019). Collectively, these findings show that there are some boundary conditions to the collective working memory effect. It should be noted that all of these findings were obtained in research that was conducted in a physical (e.g., school-based) context. An important boundary condition that has not yet been investigated is whether the nature of the learning context, physical vs. online, influences the occurrence of the collective working memory effect. In the present study, we therefore investigated the extent to which the collective working memory effect found in an offline context can also be observed in an online context.

### The collective working memory effect in an online context

The growth and popularity of online education in the past decade, accelerated by the COVID-19 pandemic, has stimulated research on effective online instruction and collaboration. According to a review by Kolm et al. (2021), there is a particular need for research on online collaboration as their results show that students are insufficiently prepared for online collaborations. Hence, this can lead to reduced engagement in collaborations and lower performance—mentioned by teachers as essential for successful online collaboration (Verstraeten et al., 2025). From this perspective, collaborating in an online environment might not yield the same benefits as working in an offline context. Specifically, online collaboration can be characterized by different—and perhaps more—transaction costs. A key feature distinguishing online collaboration from offline collaboration is the lack of face-to-face contact (DeRosa and Lepsinger, 2010). This introduces an extra difficulty compared to offline contexts by requiring learners to interpret group members’ social cues or their body language. Group members are constantly busy understanding each other’s beliefs or actions and this appears more difficult in a virtual environment (Boughzala et al., 2012). Furthermore, the lack of face-to-face contact can lead to decreased accountability which, in turn, can lead to social loafing (Boughzala et al., 2012). Moreover, the wide variation in learners’ experience, proficiency, and familiarity with online collaboration tools like Microsoft Teams may further increase cognitive demands in online collaborative environments. Of course, it is also possible that online collaboration is successful, for example if collaborators sense feelings of social presence as if they were experiencing the immediacy, warmth, and interpersonal rapport that is present in physical communication (Boughzala et al., 2012). Also, if asked to contribute solutions to a problem-solving task, the lack of face-to-face contact might reduce social barriers to express yourself and share your solution(s) with the group. Overall, the extent to which potential benefits and drawbacks of online collaboration impact the collective working memory effect remains to be systematically investigated.

The only experimental study to date investigating both social setting (individual vs. collaboration) and task complexity (low vs. high) in an online environment is that of Almaatouq et al. (2021). In their study, students were asked to solve five room assignment tasks of varying complexity. Students either worked individually or together in groups of three in an online environment. While students in the group condition did not see or speak to each other, they could send each other chat messages during the collaborative assignment. Results showed that groups outperformed even the most efficient individuals on high-complexity tasks, but not on low-complexity tasks. The results are consistent with those previously described by Kirschner et al. (2011) in their research in an offline context. Almaatouq et al.’s (2021) findings are promising and suggest that online collaboration can indeed be effective on high-complexity tasks. Their study, however, focused only on online communication through chat messaging which offers a restricted collaborative experience. In fact, this might have limited the transaction costs that are typically encountered in perceptually richer online collaborative settings. For example, in the Almaatouq et al. (2021) study, learners did not see their collaborators and therefore did not need to interpret their collaborator’s social cues. This raises the question whether the collaborative task in their study was actually experienced as difficult—something not measured in their study—and makes their study less comparable to an offline collaborative environment. The present study addresses these gaps by investigating to what extent the collective working memory effect obtained in an offline environment transfers to an online setting.

### The present study

In the current study, the effects of task complexity and social setting on cognitive load and task performance are examined in an online higher education environment. Students solved a series of high-complexity or low-complexity room assignment tasks (cf. Almaatouq et al., 2021) either individually or in groups, with group members having the opportunity to communicate verbally and non-verbally with each other during the collaboration.

Based on CLT and prior research findings, a transferability hypothesis—that is, the collective working memory effect found in offline contexts transfer to online contexts—predicts that students who work individually achieve higher task performance and report lower cognitive load on low-complexity tasks than students collaborating in a group (cf. Kirschner et al., 2011). Conversely, on high-complexity tasks students collaborating in groups are expected to obtain higher performance and report lower cognitive load than students working individually (cf. Almaatouq et al., 2021; Kirschner et al., 2011). Under the context-dependency hypothesis—that is, the distinct nature of the online setting introduces different cognitive demands compared to offline contexts—the prediction is that the patterns observed in offline contexts are absent or even reverse in an online context: on low-complexity tasks, individuals will have equal or lower task performance and equal or higher cognitive load compared to those who collaborate. On high-complexity tasks, students who collaborate will obtain equal or lower task performance and report equal or higher cognitive load than those working individually. To enable direct comparisons to prior research (e.g., Kirschner et al., 2011) regarding cognitive load, we used the single-item mental effort rating scale developed by Paas (1992) and took this up in our hypotheses. To better understand the constraints that students experience in offline and online social settings, we also asked participants to report their mental effort using a multi-item measure of cognitive load that distinguishes between intrinsic, extraneous, and germane load (Tremblay et al., 2018). However, due to the exploratory nature of this measure, we did not formulate specific hypotheses regarding these components.

## Method

### Participants

An *a priori* power analysis for a 2 × 2 ANOVA with four groups was conducted in G*Power, using an alpha level of 0.05 and a power of 0.80. The analysis indicated that a total required sample size of 151 to detect a medium-sized effect. Therefore, we opted for a sample size of 38 participants per condition (*N* = 152). However, due to practical constraints in achieving this sample size, we were able to test 86 participants, which is comparable to prior research on the collective working memory effect (e.g., Kirschner et al., 2011). Participants were recruited from the Psychology program at a large university in the Netherlands and the researchers’ social circles. Of all 86 participants, one participant did not complete the experiment and therefore was excluded from the analyses. Of the remaining 85 participants, 30 participants identified as male, 54 as female and 1 participant identified as “other”. The average age of the participants was 23.05 years (SD = 2.34). Most of the participants were higher education students (65% studied at a university, 27% studied at an applied university), 7% studied in vocational education, and only 1% were high school students. The experiment was approved by the ethics committee of the university where it was conducted.

### Design

The experimental study used a 2 × 2 design with two between-subjects factors: social setting (individual or group) and task complexity (low-complexity or high-complexity). Participants were randomly assigned to one of the four experimental conditions. There were 21 participants in the individual/low-complexity condition, 22 in the individual/high-complexity condition, 21 in the group/low-complexity condition, and 21 in the group/high-complexity condition. Following prior collaborative learning research, participants collaborated in groups of three in the group conditions, conform studies by Almaatouq et al. (2021, 2023), Kirschner et al. (2011), and Zambrano et al. (2019). The rationale for choosing groups of three also follows from prior work examining the effects of group size on student performance, showing that the largest effects are found for groups of 3–5 students (for a meta-analysis, see Tian and Zheng, 2024).

### Materials

#### Room assignment task

**Figure 1:**
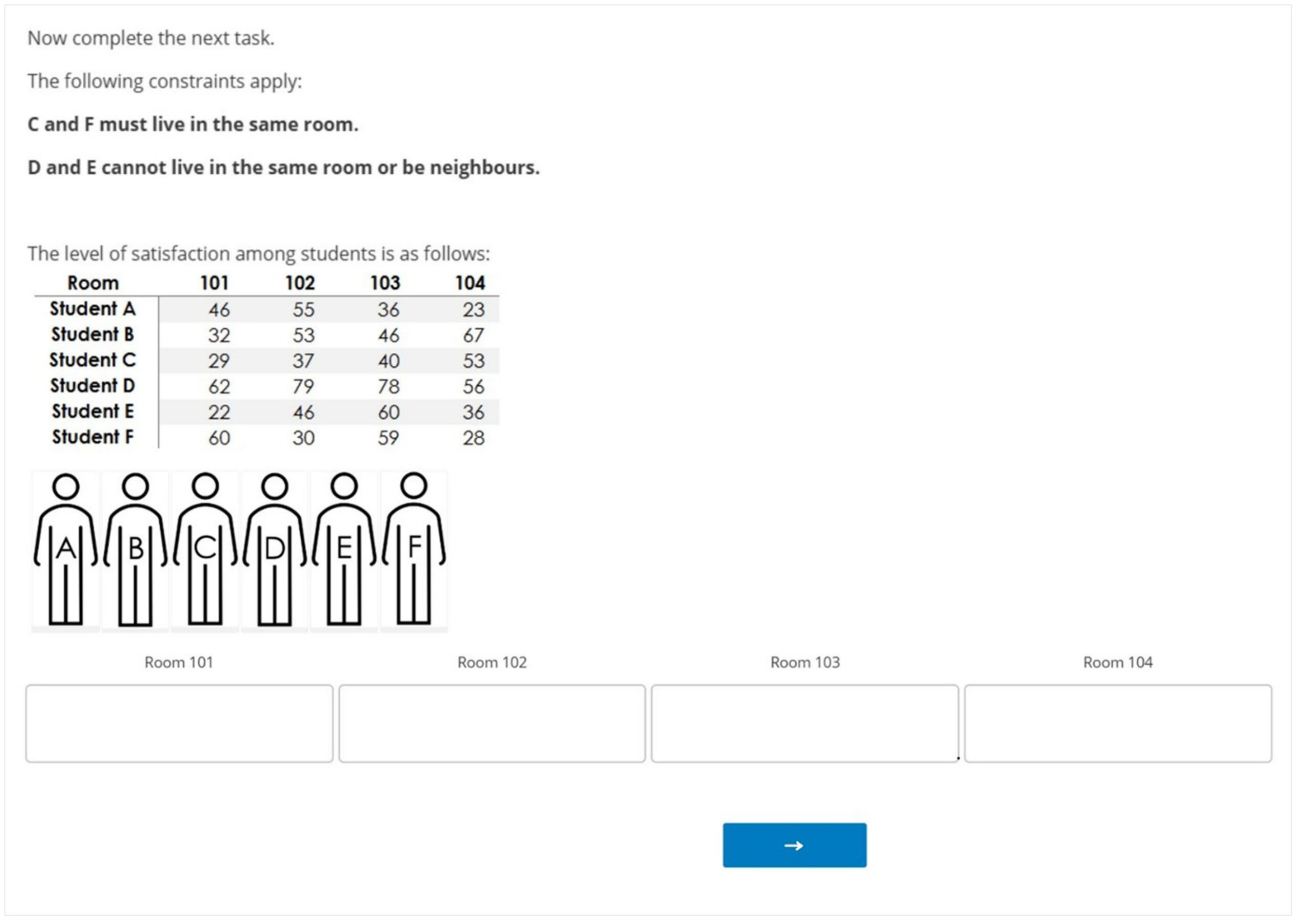
Example of a room assignment task exercise.

In all conditions, participants completed a set of problem-solving tasks. These tasks featured room assignment tasks developed by Almaatouq et al. (2021), characterized as a type of Constraint Satisfaction Optimization Problems (CSOP) where problems need to be solved taking into account specific constraints. Each task in the room assignment task involved allocating *N* students into *M* rooms to optimize utility while adhering to *Q* constraints (see for an example Figure 1). Successfully completing the task requires that each information element is considered. It is not necessary for participants to have specialized skills, which excludes possible influences of prior knowledge (Almaatouq et al., 2021). At the same time, changing the parameters *N*, *M*, and *Q* to represent either difficult or easy tasks directly correspond to the experienced complexity of the participants (Almaatouq et al., 2021), making this a suitable task to examine the effects of task complexity. We adapted the original materials from Almaatouq et al. (2021) to enable presentation in Qualtrics while attempting to retain the functionalities of the original experiment. Our Qualtrics environment enabled participants to drag and drop icons of virtual students into rooms.

#### Task complexity

Following Almaatouq et al. (2021), task complexity was adjusted by manipulating the settings of the room assignment task to create low-complexity and high-complexity tasks. This was achieved by varying the number of students (*N*), rooms (*M*), and constraints (*Q*) of the room assignment task. The low-complexity tasks required participants to assign six students to four rooms while adhering to two constraints. The high-complexity tasks required participants to assign eight students to six rooms while adhering to eight constraints. The number of tasks varied across complexity conditions to keep time on task comparable, with six tasks presented in the low-complexity condition and three tasks in the high-complexity condition.

### Measures

#### Demographic variables

The following demographic data were collected: age, gender, and educational level. Participants indicated their age in years in a text field. They reported their gender as *male*, *female*, *other*, or *prefer not to say*. For educational level participants indicated whether they had completed *secondary education*, *vocational education*, *higher professional education*, or *academic education.*

#### Task performance

Task performance for the room assignment task was calculated using the automatic scoring procedure by Almaatouq et al. (2021). Participants’ performance was based on the objective of the task to optimize utility by allocating students to rooms while adhering to the given constraints. Participants were instructed to try to achieve the highest possible score by allocating students to rooms. The maximum total score for the six low-complexity tasks was 2084, which was the sum of the scores of all six tasks. For the three high-complexity tasks, the total score was 1816, which was the sum of the scores of all three tasks. As in the Almaatouq et al. (2021) study, for each constraint violation 100 points were deducted from the total score. The minimum score on all tasks was zero. To enable comparisons across the low- and high-complexity tasks, the task performance score was calculated as a ratio score by dividing the total score by the maximum score.

#### Total cognitive load

As a measure of total cognitive load we used the self-report mental effort scale developed by Paas (1992). Participants were asked to rate the amount of mental effort they invested in the task on a nine-point Likert scale, ranging from 1 (“Very, very low mental effort”) to 9 (“Very, very high mental effort”).

#### Specific cognitive load

In addition to the single-item measure reflecting the total cognitive load, we used a multi-item measure to measure the three different types of cognitive load. For this, we used the multi-item scale developed by Tremblay et al. (2018), which is an adapted version of the cognitive load scale by Leppink et al. (2013). This 13-item self-report scale from Tremblay et al. contains four items measuring intrinsic cognitive load (sample item: “The content of this activity was very complex”), four items measuring extraneous cognitive load (sample item: “The explanations and instructions in this activity were very unclear”) and five items measuring germane cognitive load (sample item: “This activity really enhanced my knowledge and understanding of how to deal with the problems covered.”). Responses were measured on an 11-point Likert scale ranging from 0 (“not at all”) to 10 (“completely the case”). In the present sample, the Cronbach’s alpha coefficients for intrinsic load, extraneous load and germane load were 0.90, 0.85 and 0.95, respectively.

#### Efficiency

To enable comparisons that align as closely as possible to previous research, we also calculated instructional efficiency as an additional measure. This measure of efficiency was obtained by combining task performance and total cognitive load, following the approach by Kirschner et al. (2011). That is, instructional efficiency was calculated by standardizing each participant’s performance scores and perceived cognitive load on the single-item Paas scale. This was done by subtracting the mean from each score and dividing the result by the overall standard deviation, resulting in *z*-scores for cognitive load (*R*) and performance (*P*). An efficiency score (*E*) was then calculated for each participant using the formula: *E* = [(*P* − *R*)/√2]. An instructional condition is efficient if it demands less cognitive effort while maintaining an equal or higher performance. In contrast, an instructional condition is less efficient if it demands a higher cognitive effort while equal or lower performance is obtained.

### Procedure

The experiment took place in a quiet room without distractions within the University’s Behavioral Lab or externally at participant’s homes. Participants were first given a brief introduction and general explanation of the task and gave informed consent. Subsequently, participants completed the demographics questionnaire. Next, participants started the experiment in their assigned experimental condition. In the individual conditions participants completed the room assignment tasks individually. In the group conditions, participants were assigned to their group and each group received a link to an online environment for collaboration via Microsoft Teams. For the online collaboration, participants were instructed to enable audio and camera. They were instructed to work together as a team to arrive at one answer and to only communicate about task-related topics to minimize off-task interactions. Students were not allowed to take notes to prevent information from being offloaded from working memory (cf. Kirschner et al., 2011). After all room assignment tasks were completed, the perceived cognitive load was measured, respectively, using the 1-item mental effort measure (Paas, 1992) and subsequently the multi-item cognitive load measurement (Tremblay et al., 2018) was administered. Finally, all participants were sent to a debriefing page explaining the study’s purpose. The total experiment lasted about 45 min.

## Results

All analyses were conducted in SPSS version 31, with the significance level set at 0.05. Partial eta squared (*η*^2^*p*) was used as the effect size measure, with values of 0.010, 0.080, and 0.140 corresponding to small, medium, and large effects, respectively (Adams and Conway, 2014; Cohen, 1988). Furthermore, observations of performance and total cognitive load from Task 6 was excluded from all analyses, because the individual condition represented an outlying underperformance on Task 6 (*M* = 0.62, SD = 0.41) compared to Tasks 1–5 (*M* = 0.94, SD = 0.12).

### Performance and total cognitive load

We conducted a 2 × 2 multivariate analysis of variance (MANOVA) with task complexity (low vs. high) and collaborative learning (individual vs. group) as independent variables on performance and total cognitive load. Box’s *M* test was significant, *p* < 0.001; therefore, results were interpreted using Pillai’s Trace, which provides a more robust statistic. Levene’s tests indicated inequality of variances for performance, *p* < 0.001. However, because group sizes were equal, the MANOVA was considered robust. Table 1 displays the means and standard deviations for performance and total cognitive load per condition.

**Table 1:** Means and standard deviations (SD) for performance and total cognitive load in the individual and collaborative learning conditions as a function of task complexity.

	Task complexity	Performance		Total cognitive load	
		Mean	SD	Mean	SD
Individual learning	Low	0.94	0.05	3.11	1.60
	High	0.94	0.06	5.02	1.36
Collaborative learning	Low	1.00	0.00	3.34	1.44
	High	0.90	0.09	4.62	1.90

The analysis revealed a non-significant multivariate main effect for collaborative learning, *F* (2, 80) = 0.35, *p* = 0.708, *η*^2^*p* = 0.009. This indicates that, overall, learning individually and learning in a group yielded no significant differences in performance and total cognitive load. In contrast, there was a large significant multivariate main effect of task complexity, *F* (2, 80) = 16.92, *p* < 0.001, *η*^2^*p* = 0.297. This shows that there were significant differences in performance and/or total cognitive load between low- and high-complexity tasks. Also, there was a medium-sized significant multivariate interaction between collaborative learning and task complexity, *F* (2, 80) = 7.15, *p* = 0.001, *η*^2^*p* = 0.152. This indicates that task complexity differentially influenced performance and/or total cognitive load for learners who studied individually versus those who studied in a group.

To determine which dependent variables contributed to the multivariate effects, separate 2 (collaborative learning: individual vs. group) × 2 (task complexity: low vs. high) univariate ANOVAs were conducted for performance and total cognitive load. For task complexity, large significant main effects emerged for both performance, *F* (1, 81) = 15.16, *p* < 0.001, *η*^2^*p* = 0.158, and total cognitive load, *F* (1, 81) = 21.37, *p* < 0.001, *η*^2^*p* = 0.209. Participants who completed low-complexity tasks showed higher performance (*M* = 0.97, SD = 0.05) and lower total cognitive load (*M* = 3.22, SD = 1.51) than participants completing high-complexity tasks (performance: *M* = 0.92, SD = 0.07; total cognitive load: *M* = 4.82, SD = 1.64). Regarding the interaction between collaborative learning and task complexity, there was a large significant interaction effect for performance, *F* (1, 81) = 13.12, *p* < 0.001, *η*^2^*p* = 0.139.

To follow up on the significant interaction, we conducted pairwise comparisons using Bonferroni adjustments. These analyses show that individual and collaborative learning conditions did not differ significantly on high complexity tasks, *p* = 0.052. However, on low complexity tasks the collaborative learning condition scored significantly higher than the individual learning condition (*p* = 0.002). This indicates that group work improved performance for low-complexity tasks but reduced performance for high-complexity tasks. For total cognitive load no significant interaction was observed *F* (1, 81) = 2.11, *p* = 0.362, *η*^2^*p* = 0.010, indicating that individual and collaborative learning conditions reported a similar amount of total cognitive load in low and high task complexity tasks.

### Types of cognitive load

To investigate the effects of task complexity (low vs. high) and collaborative learning condition (individual vs. group) on the three different types of cognitive load (intrinsic, extraneous, and germane cognitive load), we conducted a 2 × 2 multivariate analysis of variance (MANOVA). Box’s *M* test of equality of covariance matrices was significant, *p* = 0.005; therefore, results were interpreted using Pillai’s Trace. Levene’s tests indicated that the homogeneity-of-variance assumption was violated for intrinsic (*p* = 0.002) and extraneous (*p* = 0.014) cognitive load, but because group sizes were equal, the MANOVA was considered robust. Table 2 displays the means and standard deviations regarding the three types of cognitive load per condition.

**Table 2:** Means and standard deviations (SD) for intrinsic, extraneous, and germane cognitive load as a function of collaborative setting (individual vs. collaborative) and task complexity.

	Task complexity	Intrinsic		Extraneous		Germane	
		Mean	SD	Mean	SD	Mean	SD
Individual	Low	1.98	1.27	0.42	0.61	1.92	1.40
	High	3.40	1.36	1.20	1.22	3.17	2.12
Collaborative	Low	2.03	0.86	0.62	0.63	2.66	1.72
	High	3.68	1.84	1.17	1.03	3.07	1.88
Total	Low	2.00	1.08	0.52	0.62	2.28	1.59
	High	3.54	1.61	1.19	1.12	3.12	1.98

The MANOVA revealed an insignificant multivariate main effect of collaborative learning, *F* (3, 79) = 0.25, *p* = 0.861, *η*^2^*p* = 0.009. This indicates that learners who studied individually did not significantly differ from those who studied in a group on intrinsic, extraneous and/or germane cognitive load. However, there was a large, significant multivariate main effect of task complexity, *F* (3, 79) = 8.77, *p* < 0.001, *η*^2^*p* = 0.250. This indicates that scores on intrinsic, extraneous and/or germane cognitive load differed for tasks with low and high complexity. There was no significant interaction between collaborative learning and task complexity, *F* (3, 79) = 0.97, *p* = 0.410, *η*^2^*p* = 0.036.

Separate 2 (collaborative learning: individual vs. group) × 2 (task complexity: low vs. high) follow-up ANOVAs were conducted on each type of cognitive load. Results showed that task complexity significantly affected all three types of cognitive load: intrinsic load, *F* (1, 81) = 26.28, *p* < 0.001, *η*^2^*p* = 0.245 (large effect); extraneous load, *F* (1, 81) = 11.45, *p* = 0.001, *η*^2^*p* = 0.124 (large effect); and germane load, *F* (1, 81) = 4.94, *p* = 0.036, *η*^2^*p* = 0.053 (medium effect). As can be seen in Table 2, learners reported higher scores on all types of cognitive load on high-complexity compared to low-complexity tasks. No significant main effect of collaborative learning and no significant interaction were found (*p*s > 0.05).

### Efficiency

A 2 × 2 analysis of variance (ANOVA) was conducted to investigate the effects of task complexity (low vs. high) and collaborative learning (individual vs. group) on instructional efficiency. Levene’s test of equality of error variances was significant, *p* = 0.004. Because group sizes were equal, the ANOVA was considered robust to this violation. Table 3 displays the means and standard deviations regarding the efficiency scores per condition.

**Table 3:** Efficiency means and standard deviations (SD) as a function of collaborative setting and task complexity.

	Task complexity	Mean	SD
Individual learning	Low	0.33	0.94
	High	−0.48	0.85
Collaborative learning	Low	0.83	0.58
	High	−0.69	1.19

There was a large, significant main effect of task complexity on efficiency, *F* (1, 81) = 34.60, *p* < 0.001, *η*^2^*p* = 0.296. This effect shows that high-complexity tasks were completed less efficiently (*M* = −0.59, SD = 1.03) than low-complexity (*M* = 0.57, SD = 0.82) tasks. The main effect of collaborative learning condition was not significant, *F* (1, 81) = 0.51, *p* = 0.476, *η*^2^*p* = 0.006. This indicates that learning individually (*M* = −0.07, SD = 0.98) or collaboratively (*M* = 0.07, SD = 1.20) did not affect the efficiency while solving the tasks. There was no significant interaction between task complexity and collaborative learning on efficiency, *F* (1, 81) = 3.26, *p* = 0.075, *η*^2^*p* = 0.039. As can be seen in Table 3, efficiency scores were similar for individual and collaborative learners on low and high-complexity tasks.

## Discussion

The present study shows that solving low-complexity tasks benefits the performance of learners compared to solving high-complexity tasks, irrespective of whether they work individually or in a group. These effects were observed across measures of task performance, cognitive load and efficiency, which indicates that the room assignment task we used indeed captured tasks of low and high complexity, enabling us to systematically examine how task complexity influences performance and subjective experiences. Of more relevance in light of the goal of this study, is that we found a significant interaction effect between task complexity and learning setting. Surprisingly, in contrast to the transferability hypothesis, participants who worked in groups had higher task performance and were more efficient when working on *low*-complexity tasks than participants who worked individually. These findings are opposite to the collective working memory effect originally reported by Kirschner et al. (2011) where collaboration on high-complexity tasks was more efficient than on low-complexity tasks. Moreover, they are in contrast to prior findings by Almaatouq et al. (2021) who found that groups outperform individuals on complex instead of on easy tasks. Instead, our findings are consistent with previous research demonstrating that there are boundary conditions to the collective working memory effect (e.g., Retnowati et al., 2016; Zambrano et al., 2019).

Consistent with the context-dependency hypothesis, our results highlight that findings previously obtained in an offline context do not one-on-one transfer to an online context. Moreover, they suggest that even in online contexts the collective working memory effect depends on how the collaboration takes place. Apparently, the format of the interactions afforded by the technology determines whether the collaboration is successful or not: a more restricted form of communication where the interactions are limited to verbal communication via written chat messages (cf. Almaatouq et al., 2021) seems more effective than a video-based form of communication like we used in our study.

An explanation for the opposing findings in offline and online contexts could lie in that social loafing—the tendency to reduce individual effort when working in a group compared to the individual effort when working alone (Williams and Karau, 1991)—is more likely to occur in an online context than in an offline context. It is possible that the way the collaborative conditions were structured contributed to social loafing. For example, the online collaboration required that one group member shared the screen on behalf of the entire group, which might have created a situation in which the screen-sharer assumed a leading role in the collaboration, while other group members were more inclined to lean back, disengage, and to not invest substantial effort. Under this scenario, the student who takes the lead in the collaboration will likely report higher cognitive load than their group members, which becomes especially visible on high-complexity tasks given that these tasks are more demanding. Our results regarding cognitive load provide preliminary evidence for this interpretation. As can be seen in Table 2, in the collaborative conditions, there is relatively little variation in the cognitive load that participants report when working on low-complexity tasks, whereas on high-complexity tasks there is larger variation in cognitive load scores. In contrast, for participants working individually larger variation in cognitive load is seen in the low-complexity tasks than in the high-complexity tasks. The latter simply reflects individual differences that exist in perceived complexity of the task: for low-complexity tasks the cognitive demands induced by the task can vary from one person to the other, whereas for high-complexity tasks most students experience the tasks as cognitively demanding because they do not have other group’ members to fall back on. During offline collaboration, social loafing might have less influence, due to stronger social ties and reduced anonymity (Shiue et al., 2010). This explanation remains speculative, however, as the current study did not gather data on experienced social loafing. Future research is needed to establish whether and how social loafing may have influenced the findings of our study, and to determine the role of online and offline settings for the occurrence of social loafing among group members.

Another explanation might be that online collaboration is inherently more demanding than offline collaboration. Essentially, online collaboration involves different communication and interaction dynamics due to reduced nonverbal cues and increased anonymity (Lieberman and Schroeder, 2020). Additionally, technical challenges such as the requirement to share the screen can increase extraneous cognitive load for learners. It is possible that such demands lead to lower levels of participation, which in turn results in lower performance and efficiency. While such an interpretation is in line with our findings that collaboration was more beneficial in low-complexity tasks, our findings show similar levels of cognitive load across groups and individuals, with comparable levels of intrinsic, extraneous and germane load. It remains unknown from our study why no differences were found between collaborative and individual learning on three types of cognitive load. It is conceivable that each type of cognitive load was altered for different reasons, eventually resulting in comparable cognitive load ratings. For example, participants in the individual condition may have experienced extraneous cognitive load because they had to maintain all relevant elements in their own working memory. Participants in the collaborative condition may have experienced reduced extraneous cognitive load by distributing this working memory demand across group members. However, collaboration may also have introduced additional extraneous load due to coordination and transaction costs within the group, washing out the potential benefits distributing the working memory load over group members and resulting in a higher extraneous cognitive load than anticipated. Future research could examine the feasibility of such an explanation by more precisely investigating how collaboration setting impacts students’ experienced cognitive demands and mental effort ratings. Furthermore, it is important to define which factors are especially influential, considering that we did not find benefits of collaboration in an online collaboration setting in which students interacted via live video whereas Almaatouq et al. (2021) did find benefits of online collaboration versus individual problem solving in a setting that only allowed digital text-based communication.

### Limitations and future research

While we were able to demonstrate differences in task complexity, distinguishing between low- and high-complexity tasks, the high-complexity tasks were not perceived as extremely complex. Participants indicated an average cognitive load score of around 5 out of 9 when working on high-complexity tasks. Even though the high-complexity task that we used required participants to assign nine students to six rooms, with eight constraints, more complex alternatives are possible. For example, in the Almaatouq et al. (2023) study, one of the tasks had very high complexity featuring a room assignment task in which 13 persons had to be assigned to 18 rooms using 18 constraints. Future research could investigate whether our findings are tenable under more extreme complexity conditions, for instance by comparing very low, moderate and very high complexity tasks.

The current study follows-up on prior work that has empirically demonstrated the collective working memory effect (Kirschner et al., 2011). In the Kirschner et al. (2011) study, four groups of students were included: a group of students working independently on an easy task; a group of students working independently on a difficult task; a group of three students working together on an easy task; and a group of three students working independently on a difficult task. Our study followed a similar design, with groups working together online instead of offline. An important limitation of this design is, however, that it creates variations between, but not within groups. An approach that circumvents this issue was reported by Almaatouq et al. (2021) who compared interacting groups—individuals who together form a group and interactively solve the tasks—to nominal groups—individuals who individually perform the task and together form a group of non-interacting individuals. The nominal group serves as a valuable benchmark to the interacting group, as they do reflect the intellectual resources that would be present in the group. With this approach, group interaction and not the fact that more resources are available is the deciding factor that an interacting group outperforms a nominal group (Almaatouq et al., 2021). Future studies on the collective working memory effect could adopt Almaatouq et al.’s (2021) approach to compare groups to nominal groups instead of comparing groups to individuals.

Another avenue for future research lies in advancing the efficiency measures for group performance. Many studies in cognitive psychology use a mental effort efficiency measurement: performance relative to the mental effort that was experienced by the students (e.g., Kirschner et al., 2011). Others, such as Almaatouq et al. (2021), measured efficiency as the relation between the performance of students and the time they took to solve the tasks. In the current study, time on task for completion of the problem-solving tasks was not measured. Nevertheless, time is, in addition to performance and cognitive load, an important metric for the efficiency of learning, especially for the tasks used in the current study. For example, Almaatouq et al. (2021) found that while performance was relatively comparable with students on difficult tasks scoring 10 % points lower than on easy tasks, they spent about three times as much time on solving the tasks, pointing to drastically lower efficiency. For future research, it would be interesting to compare the three outcomes—performance, time on task and cognitive load—to construct a three-dimensional efficiency measure which can provide even more insight in how students and groups performed on collaborative learning tasks and to provide a richer insight in students’ efficiency overall.

### Implications

Despite the aforementioned limitations and avenues for future research, we can tentatively formulate several educational implications based on our findings. First, it is important to make teachers and instructional designers aware that what works in offline contexts does not necessarily apply to online contexts. Second, when moving to group work, the complexity of the task that students work on should be carefully considered. The results of our study further highlight that having students collaborate on easy versus difficult tasks may not yield the same outcomes. Third, if teachers and instructional designers decide to support collaboration in an online environment, the specific features of the environment should be taken into account, as our results are confined to rich (e.g., video, talking) interaction possibilities, which is different from previous work using less rich interactive experiences (e.g., text messaging).

## Conclusion

Although the results of this study in some respects contrast with previous research (e.g., Kirschner et al., 2011), this study provides important new insights into the intricacies between task complexity and cognitive load in online learning environments. The findings show that in an online context collaboration is beneficial when working on low-complexity tasks whereas working individually is most beneficial for high-complexity tasks. Together, these findings extend previous research by highlighting that task complexity and collaboration benefits may operate differently in online learning environments than in offline learning environments. This has important implications for the design of online environments.

## Data Availability

The raw data supporting the conclusions of this article will be made available by the authors upon request; further inquiries can be directed to the corresponding author.
